# Reductive modification of genetically encoded 3-nitrotyrosine sites in alpha synuclein expressed in *E.coli*

**DOI:** 10.1016/j.redox.2019.101251

**Published:** 2019-06-10

**Authors:** Hanne R. Gerding, Christiaan Karreman, Andreas Daiber, Johannes Delp, Daniel Hammler, Martin Mex, Stefan Schildknecht, Marcel Leist

**Affiliations:** a In Vitro Toxicology and Biomedicine, Department of Biology, University of Konstanz, 78457, Konstanz, Germany; b Zentrum für Kardiologie, Johannes Gutenberg Universität Mainz, 55131, Mainz, Germany; c Research School Chemical Biology, Department of Chemistry, University of Konstanz, 78457, Konstanz, Germany

**Keywords:** Alpha synuclein, Nitration, 3-Nitrotyrosine, 3-Aminotyrosine, E.coli, ASYN, alpha synuclein, 3-AT, 3-aminotyrosine, GFP, green fluorescent protein, NCL, native chemical ligation, 3-NT, 3-nitrotyrosine, PD, Parkinsons's disease

## Abstract

Tyrosine nitration is a post-translational protein modification relevant to various pathophysiological processes. Chemical nitration procedures have been used to generate and study nitrated proteins, but these methods regularly lead to modifications at other amino acid residues. A novel strategy employs a genetic code modification that allows incorporation of 3-nitrotyrosine (3-NT) during ribosomal protein synthesis to generate a recombinant protein with defined 3-NT-sites, in the absence of other post-translational modifications. This approach was applied to study the generation and stability of the 3-NT moiety in recombinant proteins produced in *E.coli*. Nitrated alpha-synuclein (ASYN) was selected as exemplary protein, relevant in Parkinson's disease (PD). A procedure was established to obtain pure tyrosine-modified ASYN in mg amounts. However, a rapid (t_1/2_ = 0.4 h) reduction of 3-NT to 3-aminotyrosine (3-AT) was observed. When screening for potential mechanisms, we found that 3-NT can be reduced enzymatically to 3-AT, whilst biologically relevant low molecular weight reductants, such as NADPH or GSH, did not affect 3-NT. A genetic screen for *E.coli* proteins, involved in the observed 3-NT reduction, revealed the contribution of several, possibly redundant pathways. Green fluorescent protein was studied as an alternative model protein. These data confirm 3-NT reduction as a broadly-relevant pathway in *E.coli*. In conclusion, incorporation of 3-NT as a genetically-encoded non-natural amino acid allows for generation of recombinant proteins with specific nitration sites. The potential reduction of the 3-NT moiety by *E.coli,* however, requires attention to the design of the purification strategy for obtaining pure nitrated protein.

## Highlights

• 3-nitrotyrosine in proteins is reduced to 3-aminotyrosine in *E.coli*.
• 3-aminotyrosine is stable in *E.coli.*
• 3-nitrotyrosine reduction is independent of the harboring protein but affected by protein structure.

## Graphical abstract

**Figure:**
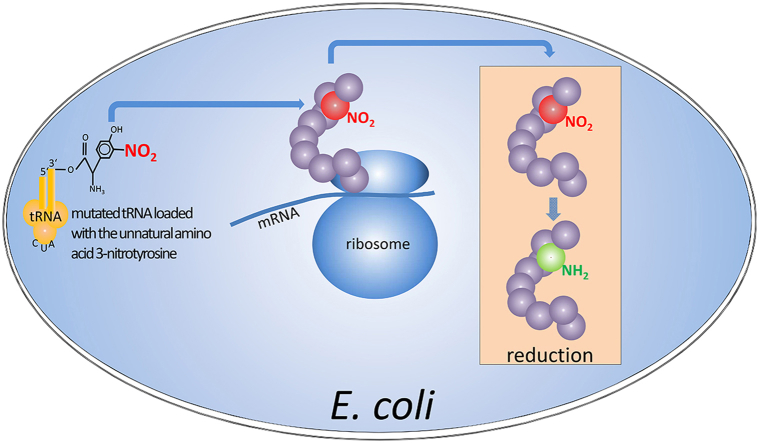

## 1 Introduction

The interaction of peroxynitrite (ONOO^−^) and its derivatives with tyrosine residues leads to the formation of 3-nitrotyrosine (3-NT) [1,2]. Under normal physiological conditions, peroxynitrite and the 3-NT modification can be involved in regulatory processes [3,4], but can also lead to detrimental consequences under conditions of oxidative stress [[5], [6], [7]]. The nitration of a tyrosine residue results in its ionization to phenolate, hereby influencing its steric and electronic properties. The nitration can thus affect the normal function of a protein [8], as exemplified by the inhibition of prostaglandin endoperoxide H_2_ synthase, prostacyclin synthase, or Mn-superoxide dismutase activity [[9], [10], [11]].

The significance of tyrosine nitration in cellular redox regulation and oxidative stress is reflected by a vast body of literature. However, almost all of these investigations are based on chemical nitration procedures, involving authentic peroxynitrite, peroxynitrite-generating compounds (such as Sin-1), or tetranitromethane [2,10,11]. Such chemical nitration procedures usually result in complex patterns of nitrated and non-nitrated residues. In addition, they also cause oxidative modifications of other amino acids, as well as covalent di-tyrosine cross-links [12]. The multiple parallel reactions limit the establishment of causal correlations between the nitration of a given tyrosine residue and a biological effect. In order to avoid these limitations, two methods for generating recombinant proteins with a defined 3-NT site in the absence of other oxidative modifications have emerged in recent years: **(1)** Native chemical ligation (NCL) allows for the extension of recombinant proteins with synthetically-derived small peptides [13,14]. For targeted insertion of a 3-NT residue into a protein, a short 3-NT-containing peptide fragment is obtained by chemical peptide synthesis. The remaining part of the protein is generated by recombinant expression and requires the presence of a C-terminal thioester. Ligation sites are typically chosen at cysteine residues. When no cysteines are present, endogenous alanine residues are mutated to cysteine and reverted back after ligation to alanine by a desulfuration step [[15], [16], [17]]. This method allows incorporation of more than one 3-NT site, but other post-translational modifications may also occur during ligation and desulfurization. **(2)** As a second method, genetic encoding of non-natural amino acids emerged in recent years as an approach for the recombinant generation of full length proteins with defined post-translational modifications [[18], [19], [20]]. In this regard, targeted co-translational insertion of 3-NT by *E.coli* has recently been established, requiring the expression of an orthogonal pair of archaeal aminoacyl-tRNA synthetase/tRNA to avoid interference with the endogenous, bacterial translation machinery [[18], [19], [20], [21]]. Genetic encoding employs the TAG DNA triplet, and the corresponding mRNA sequence (amber stop codon) that normally leads to a termination of translation [22,23]. This approach allows the formation of a recombinantly-generated protein with defined 3-NT residues at sites determined by the amber stop codon. Although pioneering work has generated a well-working and specific expression system for 3-NT containing proteins [17,21], there is still little information available on the fate of such proteins and their modifications in the producing host.

Here, alpha synuclein (ASYN), a 140 amino acid long Parkinson's disease (PD)-associated protein was chosen as relevant exemplary polypeptide [24]. The nitration of one or more of ASYN's four tyrosines has been discussed in the literature as a factor that could contribute to it acquiring a pathogenic phenotype, e.g. by influencing its membrane binding properties, its aggregation propensity, or its degradation by the proteasomal system [12,[24], [25], [26]]. To circumvent the limitations of chemical nitration, access to ASYN with defined 3-NT sites, but without other modifications, would allow insight into the causal correlation between tyrosine nitration and its influence on ASYN biology. In the present study, different proteins with genetically encoded 3-NT sites were generated and employed to study the fate of 3-NT in *E.coli*. The observations provide evidence for a reductive modification of the 3-NT group in *E.coli* that was not limited to ASYN, but also applies to other ectopically expressed proteins. The current findings need to be considered when the method of genetically encoded non-natural amino acid incorporation is applied for generating proteins with 3-NT sites.

## 2 Materials and methods

### 2.1 Bacterial strains

For the expression of ASYN full-length protein, as well as for ASYN variants carrying substitutions of one or more endogenous tyrosine residues, the *Escherichia coli* strain Tuner™ (DE3)pLysS with the genotype: F^−^ ompT hsdS_B_ (r_B_^–^ m_B_^–^) gal dcm lacY1(DE3) pLysS (Cam^R^) (gift of Prof. J. Hartig, University of Konstanz) was used. For expression of ASYN or GFP harbouring 3-NT as unnatural amino acid, the following strains were used: ***(1)*** *Echerichia coli* Tuner™ (DE3) Genotype: F– ompT hsdSB (rB– mB–) gal dcm lacY1(DE3) (gift of Prof. J. Hartig, University of Konstanz). ***(2)*** *Escherichia coli* SHuffle^®^T7. Genotype: F- lac, pro, lacI^q^/ Δ(ara-leu)7697 araD139 fhuA2 lacZ::T7 gene1 Δ(phoA)PvuII phoR ahpC* galE (or U) galK λatt::pNEB3-r1-cDsbC (Spec^R^, lacI^q^) ΔtrxB rpsL150(Str^R^) Δgor ΔmalF3 (purchased from New England BioLabs). ***(3)*** *Escherichia coli* TOP10. Genotype: F–mcrA Δ(mrr-hsdRMS-mcrBC) Φ80lacZΔM15 ΔlacX74 recA1 araD139 Δ(ara leu) 7697 galU galK rpsL (StrR) endA1 nupG.

### 2.2 Expression of non-nitrated recombinant ASYN variants

Recombinant ASYN was expressed using the *E.coli* strain Tuner (DE3)pLysS, bearing the pET11c expression vector (Novagen), carrying the gene of interest (ASYN wt and ASYN variants containing no or only one of the four natural tyrosines) under control of the T7 promotor regulated by the LacI protein. The different ASYN clones were generated via PCR with primers containing the corresponding mutations [27]. Overnight bacterial cultures (15 ml) were used to inoculate 500 ml of terrific broth (TB) medium (with ampicillin 100 μg/ml and chloramphenicol 25 μg/ml) in a 1500 ml flask. Cells were grown on a shaker (250 rpm) at 37 °C for 4 h. Expression of recombinant protein was induced by the addition of isopropyl thiogalactopyranoside (IPTG) (1 mM). After additional 2 h of growth, cells were pelleted by centrifugation (15 min, 4000 g, 4 °C), washed once with PBS, resuspended in 10–15 ml of fresh PBS, and boiled for 4 min at 100 °C. Following centrifugation at 20.000×*g* for 20 min at 4 °C, the ASYN-containing supernatant was collected and subjected to further purification.

### 2.3 Expression of ASYN or GFP with defined tyrosine nitration sites by genetically encoded non-natural amino acid technology

Recombinant proteins containing the non-natural amino acid 3-NT were expressed in the Tuner™ (DE3) and SHuffleT7 *E.coli* strains. The gene of interest (ASYN with amber stop codon at amino acid position 39 or 125; GFP with amber stop codon at amino acid position 66 or 239) was inserted into the pTxB expression vector (New England Biolabs) under control of the T7 promoter (regulated by the LacI protein). For an increased protein yield, the pEVOL vector, coding for a constitutively expressed, and an inducible, *Methanococcus jannaschii* 3-nitrotyrosyl-tRNA synthetase, was applied [28]. To further increase the efficiency of 3-NT incorporation, a second-generation amino-acyl tRNA synthetase (nitroTyr-5B) as described by Cooley was employed [29]. The *E.coli* strains expressing this second generation amino-acyl-tRNA synthethase system are indicated by an asterisk (Tuner*; Shuffle*). For protein expression, 4 × 100 ml of bacterial culture in TB medium with ampicillin and chloramphenicol were grown on a shaker over night at 37 °C. Cells were combined, pelleted by centrifugation (15 min, 4000×*g*, 4 °C), resuspended in 400 ml expression medium ((NH_4_)_2_SO_4_ 7.5 mM, NaCl 8.5 mM, KH_2_PO_4_ 22 mM, K_2_HPO_4_ 50 mM, MgSO_4_ 10 mM, CaCl_2_ 1 mg/l, FeSO_4_ 1 μg/ml, CuCl_2_ 1 μg/ml, MnSO_4_ 1 μg/ml, ZnCl_2_ 1 μg/ml, Na_2_MoO_4_ 1 μg/ml), containing IPTG (1 mM), arabinose (1%), 3-NT (2 mM), all other essential amino acids (w/o tyrosine) (0.2 g/l), ampicillin (100 μg/ml) and chloramphenicol (25 μg/ml), and distributed to 16 flasks à 25 ml. Protein expression was allowed for 4.5 h. For the assessment of the decay of the 3-NT signal in *E.coli*, bacterial cultures were treated with tetracycline (100 μg/ml) after the 4.5 h protein production phase and lysed after various time intervals. For pre-purification of heat-stable ASYN, cells were centrifuged (15 min, 4000×*g*, 4 °C), washed once with PBS, resuspended in 10–15 ml of fresh PBS, and boiled for 4 min. The lysate was centrifuged at 20.000×*g* for 20 min at 4 °C. The ASYN-containing supernatant was then stored at −80 °C, or directly subjected to further chromatography-based purification. For isolation of GFP, the boiling step was omitted, cell lysis was instead performed by sonication.

### 2.4 Chromatography affinity purification

Fifteen ml of supernatant were filtered through 0.22 μm PES-membrane syringe filters (Techno Plastic Products, Trasdingen, Switzerland) and then loaded onto a FPLC column filled with Capture Select c-tag Affinity Matrix (Invitrogen). The column was washed (10 mM Tris, pH 7.4), loaded with the sample supernatant, and washed with 10 mM Tris, pH 7.4 containing 2 M NaCl. Then, the protein was eluted by a gradient ranging from 0 to 2 M MgCl_2_ in 10 mM Tris, pH 7.4. ASYN containing fractions (3 × 5 ml) were applied to a HiPrep™ Sephadex G-25 resin desalting columns (GE healthcare) and eluted in H_2_O.

### 2.5 ASYN nitration

Purified ASYN (1 μg/200 μl of 100 mM potassium phosphate buffer, pH 7.4) was treated with authentic peroxynitrite (Merck) in the concentrations as indicated, as previously described [30]. Since peroxynitrite is provided in NaOH buffer, an equimolar amount of HCl was added to the nitration mixture to ensure maintenance of pH in the reaction sample. For this purpose, droplets of peroxynitrite (in NaOH) and HCl were carefully placed (spatially separated) on the inside of a 1.5 ml reaction tube lid, containing ASYN solution. The tube was gently closed without mixing of the two droplets, and then the sample was vigorously vortexed for optimal nitration at constant pH.

### 2.6 Western blot

Purified protein stocks (ASYN variants and GFP) were stored in H_2_O, because of a lower tendency to form covalent ASYN di- and multimers compared with storage in buffers. Samples were directly subjected to separation by a 15% SDS gel (2 μg/lane), transferred onto nitrocellulose membranes (Amersham Biosciences) and blocked with 5% milk powder in PBS-Tween (0.1%) for 1 h. Monoclonal antibodies (anti-ASYN, 1:3000, BD Bioscience; anti-3-NT, 1:250, Hycult Biotech; anti-3-NT Sigma, 1:250; anti-C-terminal amino acid sequence EPEA antibody fragment (CaptureSelect™ Biotin Anti-C-tag Conjugate, 1:1000, Thermo Fisher) were incubated over night at 4 °C. The horseradish peroxidase-conjugated secondary antibody (goat anti mouse IgG, Jackson ImmunoResearch, 1:2500), or Streptavidin-HRP (1:5000; Invitrogen) was incubated for 1 h. Protein bands were detected by a FUSION SL™ system (Peqlab, Erlangen, Germany) and quantified by ImaEva.

### 2.7 Separation of 3-NT_125_-ASYN and 3-AT_125_-ASYN

The output of FPLC-based chromatography affinity purification contains 3-NT_125_-ASYN and 3-AT_125_-ASYN. To obtain pure 3-NT-ASYN, 100 μl of SulfoLink^®^ Coupling Gel (Pierce) was washed twice with 1 ml of coupling buffer (50 mM Tris, 5 mM EDTA; pH 8.5). Anti-3-NT monoclonal antibody (HM.11, Hycult Biotech, The Netherlands), 25 μg in 100 μl coupling buffer, was combined with 50 μl of washed SulfoLink^®^ resin and incubated for 1 h at RT under constant rotation. Following binding, the antibody/SulfoLink^®^ complex was washed two times with 500 μl PBS (200 g, 2 min). For blocking of non-specific binding sites, a fresh solution of cysteine (50 mM) in coupling buffer, pH 8.5 was prepared, and added to the antibody/SulfoLink^®^ solution. Followed by 15 min of gentle mixing at RT and a subsequent incubation for 30 min without mixing, the complex was washed 2x with 1 M NaCl in H_2_O (200 g, 2 min). Following two H_2_O washing steps, the antibody/SulfoLink^®^ complex was treated twice with 500 μl of 0.1 M glycin, pH 3, followed by 2 rounds of PBS washing steps (500 μl each). The complex was then loaded with ASYN protein (60 μg/ 500 μl PBS) and incubated overnight at constant rotation at 4 °C. The antibody/3-NT-ASYN/SulfoLink^®^ complex was then washed 3x with PBS and added (in 500 μl PBS) to a 10 μm filter column (MoBiTec, M105010S). Following removal of PBS (200 g, 30 s), 3-NT-ASYN was eluted by the addition of 40 μl of 0.1 M glycin, pH 3, and centrifuged for 30 s at 200 g. This procedure was repeated up to 10 times. Each fraction was collected separately, pH was neutralized by the addition of 1 M Tris, pH 9.

### 2.8 Coomassie and silver staining

For Coomassie staining, SDS-PAGE gels were incubated in Coomassie staining solution (0.1% Coomassie brilliant blue R-250; 10% acetic acid; 40% methanol; 50% water) at 90 °C for 2 min, followed by 20 min at RT on a shaker. Gels were washed several times with water, treated with Coomassie destaining solution (10% acetic acid, 40% ethanol and 50% water), and left at RT on a shaker. For silver staining, SDS-PAGE gels were fixed for 2 h in fixing solution (50% methanol; 12% acetic acid; 0.05% formalin) and then washed 3x with washing solution (35% ethanol) for 20 min each. Gels were incubated in sensitizing solution (0.02% Na_2_S_2_O_3_) for 2 min, washed three times (5 min) in water, and stained in silver staining solution (0.2% AgNO_3_; 0.076% formalin) for 20 min. Afterwards, gels were again washed two times with water (1 min each). Gels were then incubated in developing solution (6% Na_2_CO_3_; 0.05% formalin; 0.0004% Na_2_S_2_O_3_) until bands were visible. The reaction was immediately stopped by placing the gels in stop solution (50% methanol; 12% acetic acid) for 5 min, to avoid overstaining.

### 2.9 LC tandem mass spectrometry

For identifying individual ASYN nitration sites, reversed phase liquid chromatography nanospray tandem mass spectrometry (LC-MS/MS), comprised of a Linear Trap Quadropole (LTQ) Orbitrap mass spectrometer (Thermo Fisher) and an Eksigent nano HPLC were applied. The dimensions of the reversed phase LC column were: 5 μm particle size, 100 Å pore size in a 10 cm silica capillary with an inner diameter of 75 μm (Acclaim™ PepMap™ 100C18-LC-column, Thermo Scientific). After sample injection, the column was washed for 5 min with 100% mobile phase A (0.1% formic acid) and peptides were eluted using a linear gradient of 10% mobile phase B to 40% mobile phase B within 35 min, then to 80% B in an additional 5 min, at 300 nl/min. The LTQ-Orbitrap mass spectrometer was operated in a data dependent mode in which each full MS scan (30 000 resolving power) was followed by five MS/MS scans where the five most abundant molecular ions were dynamically selected and fragmented by collision-induced dissociation (CID) using a normalized collision energy of 35% in the LTQ ion trap. Dynamic exclusion was allowed. Tandem mass spectra were analyzed by their comparison with protein databases using Mascot (Matrix Science).

### 2.10 High-resolution electrospray ionization mass spectrometry (HR-ESI-MS)

For the assessment of whole ASYN protein mass, HR-ESI-MS spectra were recorded on a Bruker Daltonics microTOF II equipped with an Agilent 1100 Series HPLC system. Samples were separated using a Macherey Nagel (Düren, Germany) EC150/2 Nucleodur 200-5 C4ec column and a binary gradient of 0.1% formic acid in water as mobile phase A and 0.1% formic acid in MeCN as mobile phase B at a flow rate of 300 μl/min. After starting with an isocratic gradient at 5% B for 5 min, fraction of B was linearly increased to 100% within 20 min. For each measurement, 20 μl of protein sample at a concentration of 0.5–1.0 μg/μl were injected. The mass spectrometric analysis was performed in positive ion mode under the following conditions: capillary voltage at 4.5 kV, nebulizer gas pressure at 0.4 bar, dry gas flow at 4 l/min and a dry temperature of 180 °C. In order to assure accuracy, sodium formate was used as internal standard in every single measurement. Processing of recorded spectra was performed using the Compass DataAnalysis software (Bruker, Bremen, Germany). Following baseline subtraction with a flatness value of 0.99 and smoothing utilizing Gauss algorithm, compound spectra were subjected to maximum entropy deconvolution to yield final spectra.

### 2.11 Amino acid analysis

Protein samples were vacuum hydrolysed in 1 ml of 6 N HCl at 110 °C overnight in vacuum hydrolysis tubes (Thermo Fisher). Subsequently, samples were freeze dried. Hydrolysed samples, or a standard amino acid mixture (100 μM each, cystine 50 μM), spiked with 30 μM 3-aminotyrosine (3-AT), were dissolved in sample dilution buffer. The amino acids were then quantified using a Sykam S433 amino acid analyzer (Sykam, Fürstenfeldbruck, Germany) using post-column derivatization with ninhydrin. Chromatography was performed using a lithium based anion exchange column loaded with spherical polystyrene resin (7 μm diameter, 10% crosslinks). Elution was performed using buffers with increasing pH and ion strength (pH 2.9 → pH 12; buffer concentration 0.12 M–0.45 M), supported by a temperature gradient. Absorbance of the reaction products was quantified at 440 nm (intermediate product; quantifies cysteine and proline) or 570 nm (quantifies all other amino acids). Amino acid concentrations were determined relative to a reference standard using the area under the peak method (v. 7 ChromStar software; SCPA, Weyhe-Leehste, Germany).

### 2.12 Transposon-mediated insertion mutagenesis

Transposon-mediated insertion mutagenesis was used to screen for *E.coli* mutants leaving the 3-NT group intact during a 3 h tetracycline chase, and to identify endogenous proteins involved in 3-NT reduction. For insertion mutagenesis, pRL27, a plasmid carrying a hyperactive Tn5 transposase gene was used [31]. The *E. coli* strain BW20767 served as donor bacterium carrying pRL27 (kanamycin resistant) and was mated with *E.coli* strain Tuner* containing the plasmids for the 3-NT-tRNA synthetase/amber suppressor tRNA_CUA_ pair and the ASYN gene containing an amber codon at amino acid position 125. The donor and the recipient strain were mixed at a ratio of 1:5, spotted on an agar plate, and cultured at 37 °C for 12 h to allow conjugational transfer of the pRL27 plasmid to the recipient cells. Tuner* clones with the integrated Tn5 cassette were selected on agar plates supplemented with kanamycin, ampicillin and chloramphenicol. The generated mutant library of 24,000 clones was transferred onto nitrocellulose and incubated for 4.5 h on filter paper with expression medium containing 3-nitrotyrosine and IPTG. The nitrocellulose with the spatially separated colonies was then transferred to new filter paper and incubated for another 3 h with tetracycline. Colonies were lysed afterwards with chloroform and lysozyme, blotted and probed with an antibody against 3-nitrotyrosine (Hycult Biotech, 1:250). To regrow the colonies, the original library was incubated at 37 °C overnight. Western Blots were used to identify the clones with a high 3-NT signal, indicating a decreased or inhibited reduction of the 3-NT to 3-AT. The integration site was then identified using inverse PCR with oligonucleotides for the Tn5 transposon. The targets identified were further confirmed by PCR-amplification and sequencing.

### 2.13 Reductive modification of 3-NT-containing peptide

A 17 amino acid-peptide (C_124_Y-(NO_2_)EMPSEEGYQDYEPEA_140_), representative of the C-terminal sequence of ASYN, with one additional cysteine residues was used (GeneCust, Luxembourg). It was synthesized so that it contained exactly one nitrated tyrosine residue (corresponding to Y_125_). This peptide had a positional nitration efficiency of 100% and did not stick to surfaces of experimental tubes, as observed with the full length ASYN protein. *Chemical reduction:* The peptide (100 μM, in water) was treated with the reducing agents for 16 h. 2-aminophenol detection was performed by the reaction with salicylaldehyde (100 μM) and AlCl_3_ (100 μM) for 90 min to obtain a fluorophore, detected at λ = 412nm_ex_/515nm_em_ [32]. 3-nitrotyrosine was detected in parallel by the assessment of its absorbance at λ = 428 nm. Calibration curves, ranging from 0 to 1000 μM were prepared in parallel with 3-NT-peptide, respectively with a 3-aminotyrosine (3-AT) containing peptide. *Enzymatic reduction:* The peptide (100 μM) was incubated with glutathione reductase (from *S.cerevisiae*; Sigma) in the presence of 1 mM NADPH, or with nitrate reductase (from *Aspergillus niger*; Sigma) in the presence of 1 mM NADPH and 100 μM FAD^+^ for 4 h. Then, 3-AT was detected by the salicylaldehyde/AlCl_3_ reaction after a 90 min incubation period at 37 °C.

### 2.14 Statistics

Spectra and blots illustrated are representative examples of at least 3 independent experiments, if not otherwise indicated. Error bars indicate data variation. Differences were tested for significance by one-way ANOVA, followed by Dunnett's post hoc test, *p* < 0.05. Statistical differences were tested using GraphPad Prism 5.0 (GraphPad Software, La Jolla, USA).

## 3 Results

### 3.1 Strengths and shortcomings of protein tyrosine nitration by chemical reactions

**Fig. 1:**
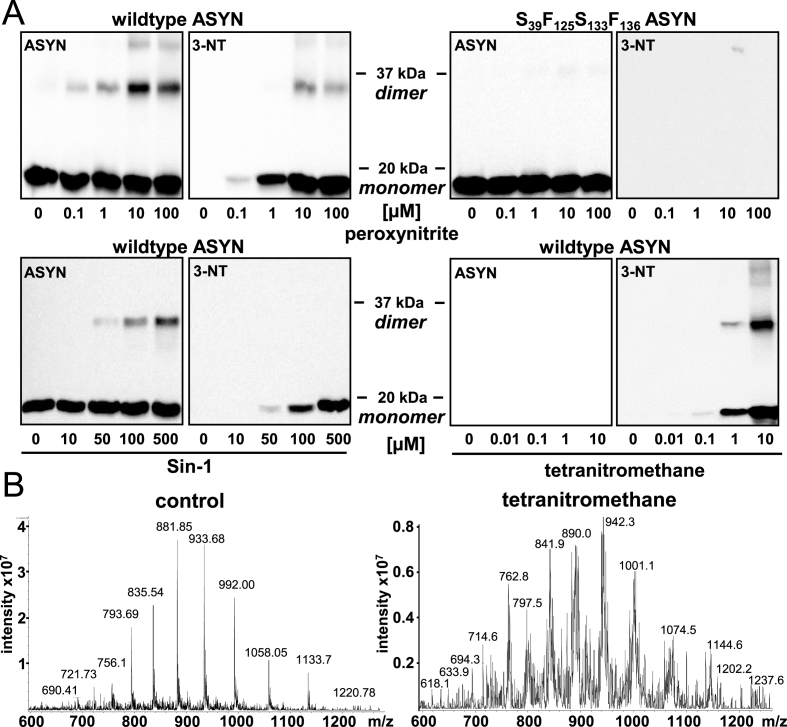
**Modification of ASYN by nitrating agents. *A)*** Wt ASYN (2 μg), respectively a mutant, in which all four endogenous tyrosine residues were substituted by serine or phenylalanine (S_39_F_125_S_133_F_136_) were treated with authentic peroxynitrite (5 min), with the peroxynitrite-generating compound Sin-1 (5 h), or with the nitrating agent tetranitromethane (5 h) at the concentrations indicated. Western blot analysis with antibodies selective for ASYN and for 3-nitrotyrosine (3-NT) indicate a concentration-dependent nitration of ASYN and a concomitant formation of a covalently linked ASYN dimer. ***B)*** ASYN (10 μg) was incubated in the presence or absence of tetranitromethane (100 μM) for 5 h and analyzed by linear ion trap mass spectrometry. On the left, the *m*/*z* (mass divided by charge) pattern of untreated, full length ASYN is illustrated. Its comparison with the *m*/*z* pattern of tetranitromethane-treated ASYN (right) shows the presence of an increased number of peaks and an overall lower peak intensity compared with the control. Western blots and MS analyses are representative for three independent experiments.

The influence of chemical nitration procedures on purified wild-type (wt) ASYN and an ASYN mutant, in which all four endogenous tyrosines were substituted by serine or phenylalanine (S_39_F_125_S_133_F_136_) was tested by the application of authentic ONOO^−^, the ONOO^−^-generating compound Sin-1, or tetranitromethane (Fig. 1A). Western blot analysis revealed that all reagents caused the formation of covalently-linked ASYN dimers and oligomers (Fig. 1A). These unwanted reaction products are most likely the consequence of dityrosine cross links, as they were not formed in the absence of tyrosine (Fig. 1A). Treatment of ASYN with increasing concentrations of ONOO^−^ and a subsequent pronase digestion and 3-NT quantification by HPLC showed that 3-NT formation reached saturation at a 3:1 M excess of ONOO^−^. Under these conditions, or with higher ONOO^−^ concentrations, the yield of 3-NT within ASYN did not exceed 25% (not shown). As expected, no antibody reactivity was observed in mutant ASYN, lacking all four tyrosine residues (Fig. 1A). To obtain an overview on the effect of chemical tyrosine nitration on the overall protein, full length ASYN was analyzed by linear ion trap mass spectrometry (MS). A Gaussian normal distribution of the mass/charge (*m*/*z*) pattern of full length ASYN was observed for the untreated protein, consistent with its molecular weight (14 460 kDa) (Fig. 1B). For instance, the peaks around *m*/*z* = 900 correspond to full length ASYN with 15–16 charges. Following nitration of an equal amount of ASYN, a global reduction in the *m*/*z* peak intensities and a drastic increase in the number of peaks was observed. This suggests that an ensemble of multiple protein species was generated upon exposure to the nitrating agent. Similar changes were observed for all other nitrating conditions (not shown). For a more detailed analysis of the oxidative modifications, occurring during the nitration procedure, nitrated ASYN was digested (Asp-N protease), the fragments were analyzed by reverse-phase liquid chromatography nanospray tandem mass spectrometry. For all peptides expected to contain tyrosine residues, variants with 3-NT instead of tyrosine were identified, and the degree of nitration increased with the concentration of the nitrating agent (not shown). In addition, multiple oxidative products of methionine, phenylalanine, and valine residues were identified. In summary, these data show that chemical nitration has *(1)* a high propensity for unwanted side reactions (oxidations, dimerization), and that *(2)* it generates a mixed ensemble of proteins with tyrosine residues being only modified in a sub-population. This situation motivated the exploration of new methods that would allow the targeted insertion of 3-NT into a recombinant protein in the absence of other oxidative modifications.

### 3.2 Site-specific incorporation of 3-NT into ASYN by genetic encoding

**Fig. 2:**
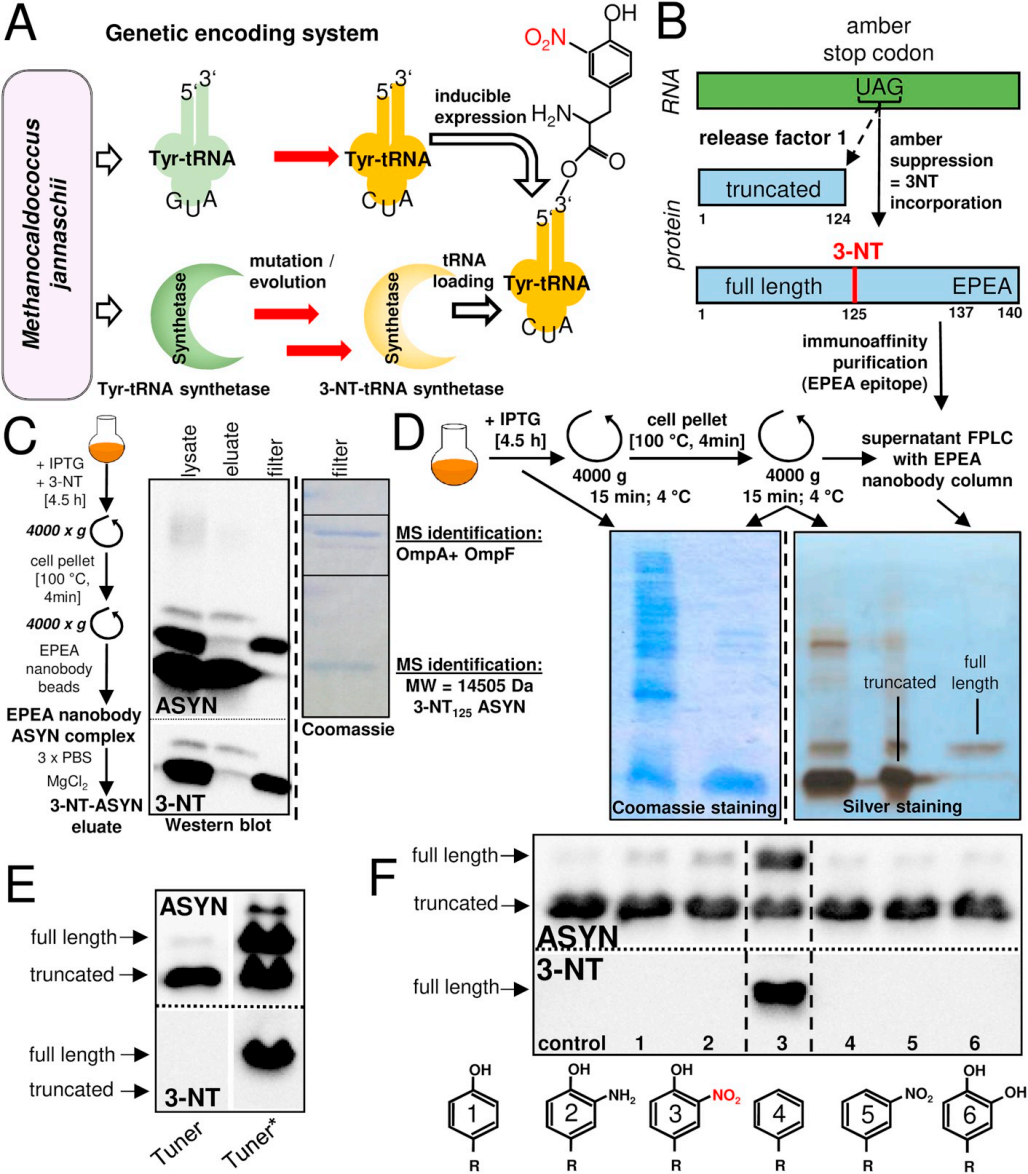
**Incorporation of 3-nitrotyrosine residues into ASYN using expanded genetic code technology. *A)*** For incorporation of 3-nitrotyrosine (3-NT) instead of tyrosine during synthesis of ASYN by *E.coli*, the amber stop codon (UAG) was re-allocated as triplet coding for 3-NT incorporation, as previously described [[18], [19], [20], [21], [22], [23]]. Mutation of archaebacterial tRNA and aminoacyl-tRNA synthetase yielded a tRNA complementary (CUA) to the amber stop codon, and a synthetase that allows the specific recognition of tRNA_CUA_ as well as the selective covalent attachment of 3-NT, but not of tyrosine. ***B)*** Mutational insertion of the amber stop codon into the DNA sequence of the target protein leads to transcripts with UAG codons at the desired site of 3-NT incorporation (green). “Amber suppression”, i.e. recognition of UAG by the 3-NT t-RNA leads to full length protein with a 3-NT group at the position desired. A truncated ASYN variant is produced, when the stop codon manifests its genuine function and binds to release factor 1. Separation of the full-length protein is achieved by a cameloid nanobody, selectively recognizing the C-terminal amino acid sequence EPEA that is present in the full length, but not in truncated protein. ***C)*** Purification of ASYN by bead-conjugated nanobodies: ASYN expression was induced in *E.coli* by isopropyl thiogalactopyranoside (1 mM) in the presence of excess 3-nitrotyrosine (3-NT) (2 mM) for 4.5 h. The cell pellet was boiled for 4 min and centrifuged to yield a supernatant fraction containing full length and truncated ASYN. To obtain a fraction of purified full length ASYN, the supernatant was incubated with bead-conjugated cameloid nanobodies, selective for the C-terminal amino acid sequence EPEA. Staining of Western blots indicated that membrane filtration allowed an enrichment of truncated ASYN in the eluate and full length protein on the filter (upper left panel). Coomassie staining of this fraction revealed the presence of at least two additional distinct protein bands that were identified by mass spectrometry analysis as a mixture of the outer membrane proteins A and F (OmpA, OmpF). ***D)*** Purification of ASYN by FPLC/nanobody column: To avoid contamination by OmpA/F, a FPLC affinity purification (nanobody-column for the specific interaction with the C-terminal ASYN sequence EPEA) was used. ***E)*** The *E.coli* strain Tuner, respectively Tuner*, carrying a high performance pEvol-enhanced expression vector, were tested with respect to the total amount of nitrated ASYN formed, and the ratio between full length and truncated ASYN. ***F)*** Tuner* was employed as ASYN producing *E.coli* strain. 3-NT (3) or the structurally related amino acids tyrosine (1), 3-aminotyrosine (2), phenylalanine (4), 3-nitrophenylalanine (5), and 3,4-dihydroxyphenylalanine (6) were supplemented during the protein synthesis period. The blots shown in C–F are representative for 4 independent experiments. (For interpretation of the references to colour in this figure legend, the reader is referred to the Web version of this article.)

For the site-specific incorporation of 3-NT into ASYN, the *E.coli* strain Tuner was modified to express an evolved *Methanococcus jannaschii* (*Mj*) 3-NT-tRNA synthetase and a cognate 3-NT-tRNA, recognizing the UAG stop codon (Fig. 2A). For the expression of recombinant protein, the pEVOL plasmid was employed. It codes for the tRNA and two copies of the 3-nitrotyrosyl-*t*-RNA synthetase, one giving rise to a constitutive level of the synthetase, and one copy that is controlled by an arabinose-inducible promoter [28]. To further boost the yield of recombinant protein, a second generation amino-acyl-tRNA synthetase, with a higher 3-NT incorporation rate, was employed (nitroTyr-5B) [29]. *E.coli* strains expressing the second generation synthetase are marked by an asterisk (Tuner*, SHuffle*). This modified system was designed to allow the incorporation of the unnatural amino acid 3-NT at an amber stop codon (UAG) instead of UAC, coding for the endogenous Y_125_ in ASYN. In the induced bacteria, the amber codon allows either the insertion of the non-natural amino acid 3-NT, or it acts in its original role as stop codon. For this reason, a mixture of full-length ASYN, carrying 3-NT at the desired site, and of a truncated version (termination of translation evoked by the amber stop codon) was produced (Fig. 2B). For the separation of the two ASYN species, a camelid nanobody, with selectivity for the C-terminal four amino acid sequence: glutamate-proline-glutamate-alanine (EPEA), was used. In initial purification attempts, the bead-conjugated nanobody-full length ASYN complex was separated from unbound truncated ASYN by means of membrane filtration. Western blotting showed that all full-length protein bound to the filter and all nitrated protein was found in this fraction, while the eluate contained truncated protein. Coomassie staining of the filter fraction confirmed the exclusive presence of full length ASYN, however, at least two additional protein bands were also detected. They were identified by MS analysis, as the outer membrane proteins A and F (Ompa, OmpF) (Fig. 2C). Such contaminations represent a problem for the application of ASYN in research projects, investigating e.g. its lipid/organelle interactions. A modified purification protocol was therefore established, involving bead-conjugated nanobody in association with a FPLC (fast protein liquid chromatography) system. This allowed the purification of full-length ASYN without significant contamination by other proteins or by truncated ASYN (Fig. 2D).

For optimization of full-length protein yield, the *E.coli* strain Tuner was employed to carry a high performance pEVOL-enhanced expression vector (Tuner*) (Fig. 2E) [28,29]. To test the specificity of the 3-NT incorporation machinery, 3-NT_125_-ASYN Tuner* cells were grown in the presence of either 3-NT or of structurally related amino acids. Full-length ASYN protein was only produced in the presence of 3-NT, while tyrosine and all other derivatives tested led to a truncation at the amber stop codon due to the failure to produce a loaded corresponding t-RNA for this site (Fig. 2F). These data suggest that accidental incorporation of tyrosine or e.g. 3-AT at the amber site did not play a significant role during ectopic protein synthesis in the *E.coli* strains applied in this work.

### 3.3 Formation of 3-aminotyrosine (3-AT) in *E.coli*

**Fig. 3:**
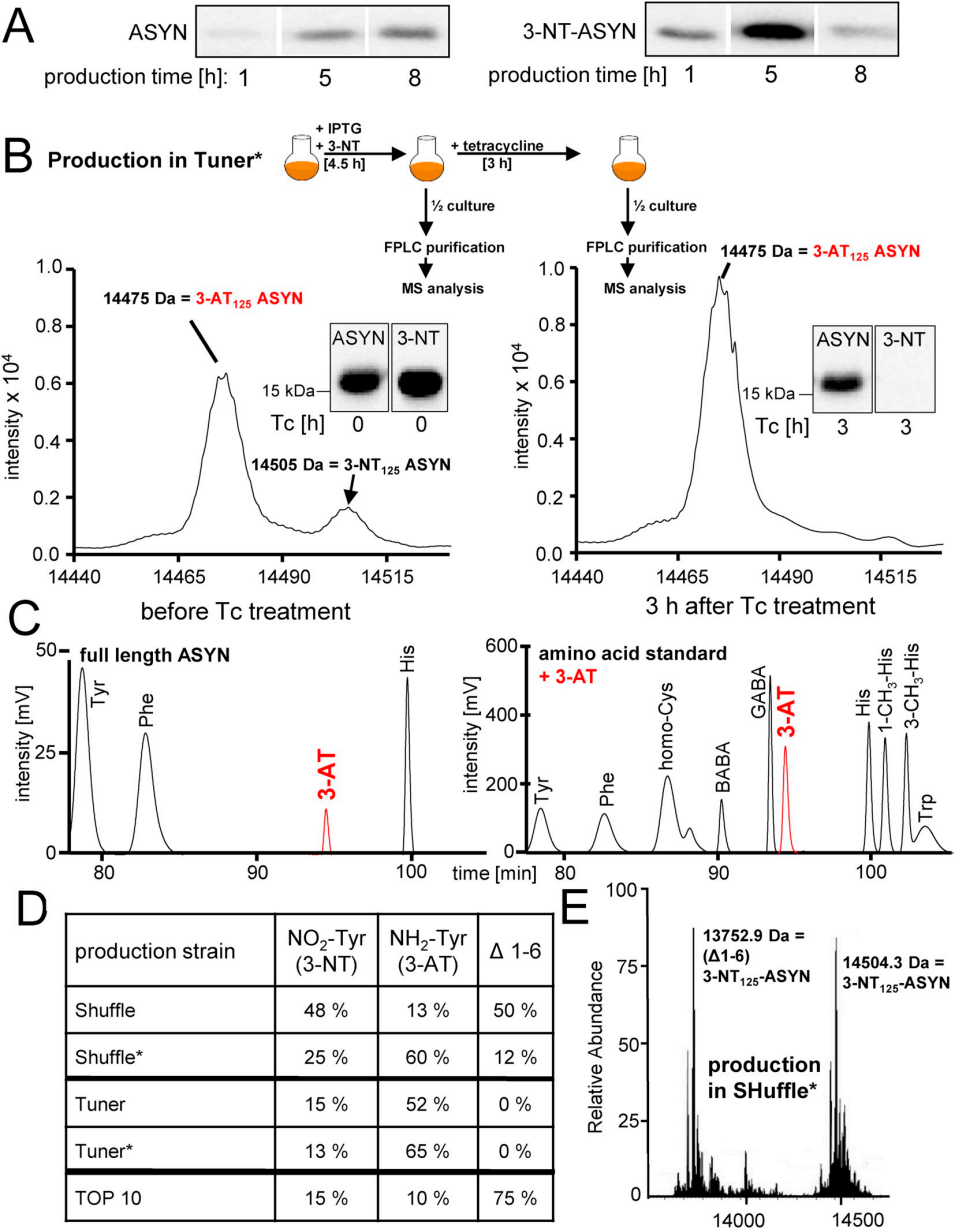
**Time-dependent loss of 3-NT in recombinantly expressed ASYN in *E.coli*. *A)*** 3-NT-ASYN was produced in *E.coli* (Tuner*) as shown in Fig. 2D. ASYN total protein and its nitration was assessed by Western blot at 1 h, 5 h, and 8 h after induction of protein synthesis. Data are representative for 10 experiments. ***B)*** Pulse-chase experiment of 3-NT-ASYN production and stability of the 3-NT group. 3-NT-ASYN was generated for 4.5 h according to the standard protocol (Fig. 2D). Then, *de novo* protein synthesis was inhibited by the addition of tetracycline (Tc) (100 μg/ml). Samples were collected at the time of tetracycline addition (0 h) and 3 h later. Western blot analysis indicated a loss of the 3-NT signal but not of ASYN over time. Analysis of the same samples by high resolution electrospray ionization (HR-ESI) mass spectrometry confirmed the loss of 3-NT and the emergence of 3-aminotyrosine (3-AT) instead. MS-spectra and Western blots are representative for 5 independent experiments. ***C)*** For an independent confirmation of the formation of 3-AT, purified 3-NT-ASYN protein (collected 3 h after Tc treatment) was completely hydrolysed (6 N HCl, 100 °C) and analyzed for its individual amino acid composition by HPLC (left). A standard amino acid mixture (100 μM each) was spiked with 3-AT (30 μM) as control (right). The spectra shown are representative for 4 independent experiments. ***D)*** High resolution electrospray ionization mass spectrometry analysis of purified ASYN, carrying 3-NT at position Tyr_125_, generated (4.5 h) in the *E.coli* strains SHuffle, Tuner, or TOP10, as well as in SHuffle* and Tuner* that both carry the pEVOL vector for higher protein yield. The table provides an overview on the respective amounts of 3-NT-ASYN, 3-AT-ASYN, as well as ASYN, lacking the N-terminal amino acids 1–6 (MDVFMK). Data are means of two independent experiments. ***E)*** Representative HR-ESI-MS deconvoluted spectrum of ASYN, carrying 3-NT at position 125, generated in Shuffle* for 4.5 h. The spectrum is representative for three independent experiments.

During experiments to optimize the output of nitrated protein, the amounts of full-length ASYN and 3-NT were assessed at different intervals following IPTG/arabinose-dependent induction of recombinant protein synthesis in Tuner*. While ASYN levels continuously increased over time, 3-NT-ASYN levels dropped after >5 h (Fig. 3A). To study the fate of nitrated ASYN in *E.coli*, tetracycline was added to block any further protein synthesis, and the remaining ASYN was analyzed 3 h later. The data showed that any substantial degradation of ASYN can be excluded for the 3 h period (Fig. 3B) and is about 50% at 24 h (Fig. 5D). A detailed analysis by high resolution electrospray ionization MS showed that 3-NT-ASYN declined within a period of 3 h. The loss of 3-NT was accompanied by a parallel rise of ASYN with a protein mass indicating the presence of a 3-AT group (Fig. 3B). To confirm 3-AT formation through an alternative methodology, 3-NT-ASYN was generated in Tuner*, *de novo* protein synthesis was interrupted by tetracycline addition for a period of 3 h, and full-length ASYN was purified and hydrolysed. Detection of the individual amino acids by HPLC confirmed the presence of 3-AT (Fig. 3C). As a third method, 3-AT formation was detected by its conversion into a fluorophore. For this, purified 3-AT-ASYN was allowed to react with salicylaldehyde and Al^3+^ and fully confirmed the observations of 3-AT formation made by mass spectrometry and amino acid analysis (not shown).

In order to investigate the strain-dependency of the observed reduction of the 3-NT group, 3-NT containing ASYN was produced in the *E.coli* strains Tuner, SHuffle (parts of its reductive system removed), TOP 10 (applied in previous studies on 3-NT incorporation) [21,33], respectively in the strains Tuner* and SHuffle*. Purified ASYN protein was investigated by HR-ESI MS (Fig. 3D). Quantitative assessment of 3-NT levels indicated that all *E.coli* strains investigated, produced 3-AT ASYN, together with 3-NT-ASYN. The SHuffle strain with a lower reducing potential produced clearly less 3-AT than Tuner (Fig. 3D). However, a detailed analysis of the ASYN protein showed a loss of the N-terminal 6 amino acids through a bacterial protease when ASYN was generated in SHuffle, SHuffle* or in TOP 10 (Fig. 3D + E). Due to the relevance of the N-terminus in ASYN biology (e.g. binding to membranes), the SHuffle and TOP 10 strains were therefore excluded for ASYN generation.

### 3.4 Factors contributing to the reduction of 3-NT to 3-AT in *E.coli*

**Fig. 4:**
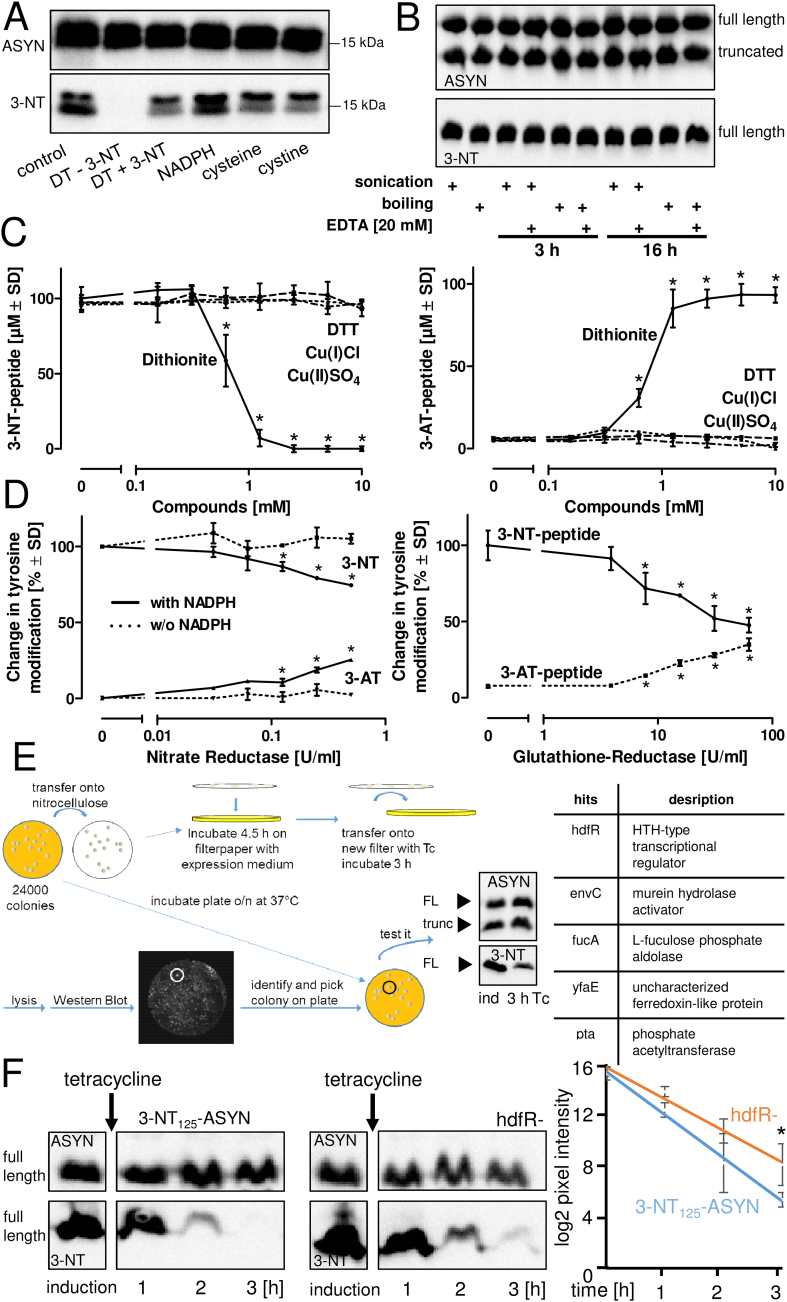
**Factors contributing to the reduction of 3-NT to 3-AT in *E.coli*. *A)*** Purified 3-NT_125_-ASYN was treated with 1 mM of different reducing reagents for 18 h at 37 °C. Dithionite (DT) was used alone (-3NT) or in the presence of free 3-NT (1 mM) ***B)*** To assess a potential influence of cell lysis on the stability of 3-NT, *E.coli,* expressing 3-NT_125_-ASYN, was either lysed by sonication or by boiling in the presence or absence of EDTA. The cell homogenates were incubated for 3 h or 16 h, 3-NT-ASYN was then purified as shown in Fig. 2D, and the amounts of full length, truncated protein, and nitration were determined by Western blot. The blots are representative for four independent experiments. ***C)*** To circumvent a mixture of 3-NT and 3-AT containing proteins, a 17 amino acid peptide, representing the C-terminal end of ASYN with one defined 3-NT site (C_124_Y-(NO_2_)EMPSEEGYQDYEPEA_140_) was synthesized and treated with the reducing compounds as indicated for 16 h. Data are means ± SD of three independent experiments. Differences were tested for significance by one-way ANOVA, followed by Dunnett's post hoc test *p < 0.05. ***D)*** The 3-NT containing peptide was incubated with glutathione reductase or with nitrate reductase for 4 h, the graphs illustrate the levels of 3-NT and 3-AT containing peptides. Data are means ± SD of three independent experiments. Differences were tested for significance by one-way ANOVA, followed by Dunnett's post hoc test *p < 0.05. ***E)*** Identification of genes contributing to the loss of 3-NT in *E.coli*: insertional mutagenesis was applied, allowing the random insertion of a transposon into the *E. coli* genome. *E. coli* clones, displaying a high residual 3-NT signal intensity after 3 h of tetracycline incubation, were selected and analyzed for the respective gene(s) affected by transposon insertion. This method allowed the identification of five target genes. ***F)*** The HTH-type transcriptional regulator (hdfR) was selected and knocked out in Tuner* to investigate stability of 3-NT_125_-ASYN in detail. Following a protein synthesis period of 4.5 h, tetracycline was added to terminate *de novo* protein synthesis. 3-NT levels in ASYN were detected over a period of 3 h and compared with 3-NT levels in ASYN generated in Tuner* without hdfR knockout. Band quantification and evaluation by trend analysis showed a significant difference in 3-NT decay between ASYN generated in the control- and the hdfR-knockout strain. Band quantifications are means ± SD of three experiments. Differences were tested for significance by two-way ANOVA (wt; hdfR-x time) followed by Tukey's post hoc test * *p* < 0.05.

In order to examine the plausibility for a reduction of the 3-NT group within a polypeptide chain, several potential non-enzymatic and enzymatic candidates were investigated [34,35]. Purified ASYN, containing 3-NT at position 125, was treated with biologically-relevant reducing agents such as NADPH or cysteine (Fig. 4A), but also with DTT, N-acetyl cysteine, or with transition metals such as iron or copper (not shown). None of them led to any reduction of 3-NT. Dithionite (DT) is a well described reductant of 3-NT and served as positive control (Fig. 4A). This feature of DT was confirmed, as the reaction of DT with 3-NT-ASYN was fully blocked by the addition of an excess of free 3-NT. To investigate a potential influence of purification conditions on the observed loss of 3-NT in ASYN, cells in the presence or absence of EDTA were lysed either by sonication, or by boiling, and then incubated at 37 °C for a period of 3 h or 16 h (Fig. 4B). These observations indicate that the observed reduction of 3-NT occurs only in intact *E.coli* and is neither an artifact evoked by the purification procedures, nor is it easily affected by isolated *E.coli* components. As mechanistic studies on full length ASYN generated in *E.coli* could yield ambiguous results due to the mixture of 3-NT- and 3-AT-containing ASYN, a peptide, representing the 17 amino acid sequence of the C-terminus of human ASYN was obtained by chemical peptide synthesis with one tyrosine (Y_125_) nitrated with an efficiency of 100%. This model peptide was treated with a series of biologically-relevant potential reductants (dithionite, GSH, NADPH, NADH, N-acetyl-cysteine, dithiothreitol, Cu(I) and Cu(II) in the presence or absence of NADH). A significant reduction of 3-NT into 3-AT was only observed with dithionite (Fig. 4C). To test a contribution of enzymatic processes in the reduction of 3-NT, nitrate reductase and glutathione reductase were chosen as models. Both enzymes allowed partial reduction of the 3-NT group in the model peptide into 3-AT (Fig. 4D) when supplied with appropriate electron donors. These observations suggest the contribution of one, or the combination of different endogenous reductases in *E.coli*, to the observed reduction of 3-NT.

**Fig. 5:**
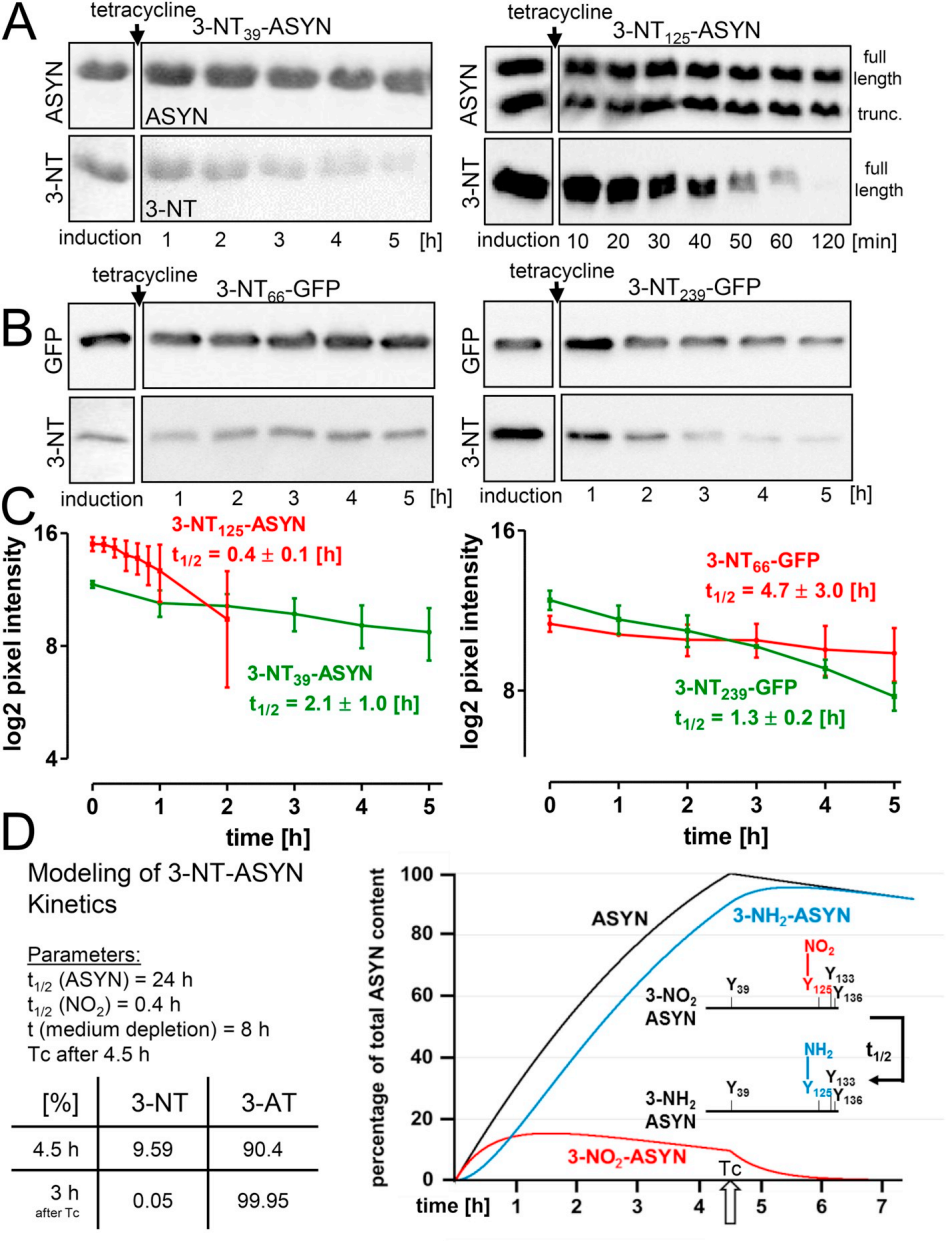
**Influence of the localization of 3-NT within a protein on its elimination. *A)*** ASYN, carrying a 3-NT group either at position Tyr_39_ or Tyr_125_ was expressed for 4.5 h in Tuner*. For analysis of 3-NT stability, protein synthesis was terminated by tetracycline (100 μg/ml), 3-NT levels were assessed thereafter in a time-dependent manner by Western blot analysis with antibodies selective for ASYN and 3-NT. Data are representative of 3 experiments. ***B)*** GFP, an alternative protein, was chosen for targeted insertion of 3-NT during protein synthesis. Tyr_66_ is located in the core of the GFP barrel, while C-terminal Tyr_239_ is exposed to the environment. ***C)*** For quantification, the 3-NT signal of ASYN and GFP is illustrated as pixel intensity (n = 3 ± SD). ***D)*** Kinetic model of 3-NT and 3-AT formation in ASYN, synthesized in Tuner*. The model is based on the experimentally detected half-life time of ASYN (24 h), and the detected half-life time of 3-NT_125_ of 0.4 h, in combination with the assumption of non-dividing *E. coli*, and a linear consumption of medium, with medium depletion after 8 h. For termination of *de novo* protein synthesis, tetracycline (Tc) is added 4.5 h after initiation of protein synthesis. The percentage of 3-NT and 3-AT is depicted graphically. Calculated values at time t = 0 (=4.5 h after induction of synthesis) and 3 h after tetracycline addition (=7.5 h after induction) are indicated in the table.

For identifying gene products that may be involved in the reduction of 3-NT to 3-AT, a transposon-mediated insertion mutagenesis screen was performed [31]. This method involves gene inactivation by random insertion of a transposon into the *E.coli* genome, combined with a selection of those clones that show a lower activity to reduce the 3-NT signal over time (Fig. 4E). Five clones were isolated, and their respective target genes, affected by transposon insertion, were analyzed. Only one of the hits (yfaE) actually coded for a protein with reductase activity. For instance, hdfR was deleted in an *E.coli* strain producing 3-NT_125_-ASYN according to the method of Datsenko [36] and this led to a significant slowdown of 3-NT reduction (Fig. 4F). It is essential to note here that random transposon-mediated insertion mutagenesis only allows for the identification of non-essential genes, as an interruption of an essential gene no longer enables colony formation. The fact that only indirect regulators were identified here indicates an essential role by the reductases involved in 3-NT reduction in *E.coli* survival.

### 3.5 Influence of the localization of 3-NT within a polypeptide on its reduction in *E.coli*

To address the question of whether localization of the 3-NT residue affects its reduction kinetics, ASYN, carrying 3-NT either at position 39 (N-terminal region), or at position 125 (C-terminal region), was expressed in Tuner*. Samples were collected at various time-points after termination of *de novo* protein synthesis by tetracycline and analyzed for their 3-NT content. A significantly faster decay of 3-NT was observed in 3-NT_125_-ASYN compared with 3-NT_39_-ASYN (Fig. 5A + C). While ASYN is considered an unstructured protein when in solution, green fluorescent protein (GFP) is characterized by its distinct three-dimensional barrel structure, and was therefore chosen as an alternative representative example for genetically encoded 3-NT insertion and the fate of 3-NT in *E.coli* [37]. GFP was expressed, carrying 3-NT either at the freely accessible C-terminal end (Y_239_), or in the center of the barrel structure (Y_66_). Tyr_239_-GFP showed a more than 3-fold faster decline of the 3-NT signal, compared with the loss of Tyr_66_-GFP nitration (Fig. 5B + C). These observations illustrate that the half-life of the 3-NT group, inserted by genetic encoding into a protein for its recombinant expression in *E.coli*, is determined by its localization. The observations further indicate that the reduction of 3-NT is not specific for ASYN, but may occur in any protein generated by non-natural amino acid incorporation technology. This unexpected 3-NT-reducing capacity of *E.coli* needs to be considered in the purification strategy of 3-NT containing proteins generated by this method (Fig. 5D).

### 3.6 Separation of 3-NT and 3-AT containing proteins

**Fig. 6:**
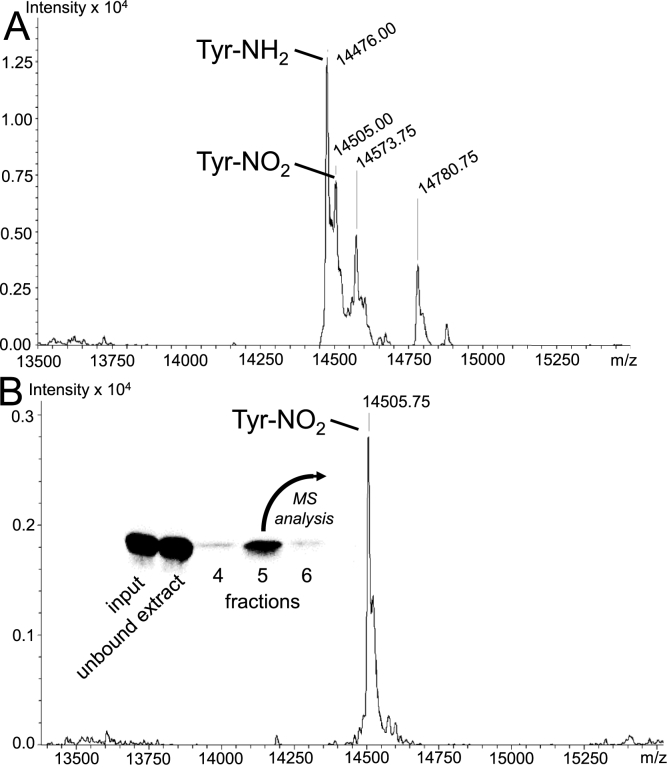
**Separation of 3-NT**_**125**_**-ASYN and 3-AT**_**125**_**-ASYN. *A)*** ASYN, carrying 3-NT at position 125 was produced in *E.coli* (Tuner*) as shown in Fig. 1, Fig. 2 μg was analyzed by high resolution electrospray ionization mass spectrometry. ***B)*** In order to allow the generation of pure 3-NT-ASYN without contamination by 3-AT-ASYN, ASYN from chromatography affinity purification (contains 3-NT_125_-ASYN and 3-AT_125_-ASYN) was subjected to a column with covalently linked anti-3-NT antibody. The Western blot illustrates ASYN staining of protein solution input to the column, the unbound extract, and different elution fractions. Protein from fraction 5 was analyzed by mass spectrometry and indicated no detectable contamination by 3-AT_125_-ASYN (n = 3).

In order to obtain a homogenous population of nitrated protein of interest, a 3-NT-selective antibody-based purification strategy was pursued. As classical immunoprecipitation leaves the antibody as contamination, the 3-NT antibody was covalently (via –SH groups) immobilized on beaded agarose. Elution of 3-NT_125_-ASYN is illustrated by Western blot (Fig. 6). For analysis of 3-NT and 3-AT groups, ASYN collected after the FPLC purification step (Fig. 6A) and after elution from the subsequent antibody-based second purification step (Fig. 6B) was analyzed by mass spectrometry. Comparison of the two spectra indicates that the antibody purification step yields a homogenous population of nitrated ASYN without detectable contaminations by 3-AT-ASYN or non-modified ASYN.

## 4 Discussion

In the present work, we explored a new method allowing the generation of proteins *(1)* with defined tyrosine nitration sites, *(2)* having no other oxidative modifications, and *(3)* allowing yields in the high microgram range. A new, unanticipated issue discovered during this work was the reduction of 3-nitrotyrosine (3-NT) to 3-aminotyrosine (3-AT) that was dependent on the location of tyrosine groups within a protein.

The co-translational incorporation of 3-NT by genetic code expansion has recently been applied for the translation of 3-NT-containing superoxide dismutase and ribonucleotide reductase [21,38]. These proof of concept studies established the basic tools enabling our work, but did not focus on the stability of the newly introduced 3-NT moiety, especially in intrinsically disordered proteins. This issue was addressed here by means of a detailed analysis of the integrity of genetically encoded 3-NT in alpha synuclein (ASYN).

The most striking observation of the present work was a time-dependent loss of the 3-NT signal, detected by Western blot and mass spectrometry. Tyrosine nitration has been considered as a relatively stable process in biological systems [[39], [40], [41]]. More recently, de-nitration activities were reported for homogenates of lung, heart, and brain, or in mitochondria [[42], [43], [44], [45]]. Most of the studies reporting the de-nitration of 3-NT are based on an antibody-dependent detection of 3-NT-containing protein epitopes. Therefore, it remained unclear whether these observations are the result of an actual de-nitration of the 3-NT group to yield tyrosine, a reduction of 3-NT to form 3-AT, or possibly the consequence of epitope alterations, independent of the 3-NT group (e.g. conformational changes or other chemical modifications). Specific enzymes, catalyzing either the de-nitration of 3-NT to tyrosine, or 3-NT reduction to 3-AT, remain unidentified.

Here we provide evidence that the decline in the 3-NT signal was caused by a reductive process yielding 3-AT. The 3-NT reduction was neither observed in lysates of homogenized *E.coli*, nor was it occurring during the lysis of bacteria or the protein isolation procedure. An erroneous translational incorporation of 3-AT or other tyrosine-derivatives can be excluded (Fig. 2F). The reduction of 3-NT was not specific for ASYN, but it was also observed with the entirely unrelated protein, green fluorescent protein (GFP) (Fig. 5B). While ASYN is considered to be an unstructured protein when in solution [46,47], GFP has a highly structured β-barrel domain [37]. 3-NT, expressed at the C-terminal end of GFP (GFP_239_), i.e. on a flexible tail at the surface of the protein, was reduced significantly faster than 3-NT located in the core of the β-barrel (GFP_66_) (Fig. 5C). These observations indicate that a major determinant of 3-NT reduction in *E.coli* is the spatial orientation of 3-NT within a protein and thus, most probably, its capacity to interact with reducing agents or enzymes. The kinetics of 3-NT decay therefore not only varies between different proteins, but even between different tyrosine residues within the same protein, as shown here for both ASYN and GFP. Based on the experimentally detected half-life times for ASYN protein and for the 3-NT group in ASYN, a mathematical model suggests maximal steady-state levels of nitrated ASYN of about 15% (Fig. 5D). These values are in agreement with our experimental observations (Fig. 3D).

The formation of 3-AT-ASYN has been reported for rotenone-exposed PC12 cells [48]. These observations indicate that the reduction of 3-NT to 3-AT also proceeds in eukaryotic cells and might modulate the functional changes evoked by cellular tyrosine nitration. It could be speculated that the formation of 3-AT might either support ASYN's pathophysiological behavior (e.g. modulation of membrane binding or aggregation), or, in contrast, could protect from the reported detrimental influences evoked by 3-NT. An identification of the 3-NT reducing components in the cell, and their targeted modulation, could hence reflect a potential intervention site to modulate ASYN pathology in the human brain.

In order to obtain a homogenous population of nitrated protein of interest, we tested the removal of 3-AT containing ASYN or GFP by the reaction of the 2-aminophenol moiety with an azide derivative of salicylaldehyde [32] and the subsequent formation of an alkyne link to agarose beads. Due to unspecific binding of ASYN to the resin, this method enabled no efficient separation of 3-NT and 3-AT containing ASYN. However, this method could serve as efficient approach for the separation of other proteins. A potential unspecific binding of the protein of interest to the complex has to be tested individually. A classical immunoprecipitation with a 3-NT-specific antibody indeed yields a 3-AT-free population of 3-NT-ASYN, but is inevitably contaminated by the heavy and light chains of the antibody used for pulldown. To circumvent these limitations, the 3-NT-antibody was covalently linked with iodoacetyl groups on beaded agarose and hereby allowed the successful separation of pure 3-NT containing ASYN (Fig. 6). The mini-columns loaded with 3-NT antibody in this study could be reused at least 5 times and yielded 3-NT-ASYN in the range of some dozens of microgram protein. The size of the columns can be adjusted accordingly to meet the respective demand for the protein of interest. During the work with ASYN, we observed an explicit tendency of this protein to attach to surfaces. To limit loss of ASYN protein during the purification steps described herein, the use of glassware or low attachment plastic tubes is highly recommended.

## 5 Conclusions

Our data indicate an efficient reduction of 3-NT groups into 3-AT by *E.coli*. This aspect requires consideration in the development of a protocol for the recombinant expression of 3-NT-containing proteins by genetic encoding techniques. To obtain a homogenous population of 3-NT containing protein, the purification strategy depends on the features of the protein of interest. The stability of ASYN allowed a boiling step that enabled *E.coli* lysis, protease inactivation, and ASYN separation from crude cellular extracts. For other proteins that do not allow boiling, an initial anion/cation exchange chromatography step for the removal of crude cell debris is necessary before the enriched protein fraction can be applied to the 3-NT antibody column.

The observation of 3-NT reduction to form 3-AT in *E.coli*, together with observations in the literature on 3-AT formation in ASYN of cells exposed to oxidative stress [48], indicate a novel mechanism that could modulate the regulatory roles of 3-NT reported in numerous cases associated with oxidative stress conditions [[5], [6], [7], [8], [9], [10], [11]]. The enzymatic or non-enzymatic processes involved in 3-NT reduction were not identified in the course of the present work and await further studies for their detailed characterization.

## Declarations of interest

None.

## References

[1] Koppenol W.H., Moreno J.J., Pryor W.A., Ischiropoulos H., Beckman J.S. (1992). Peroxynitrite, a cloaked oxidant formed by nitric oxide and superoxide. Chem. Res. Toxicol..

[2] Ischiropoulos H., Zhu L., Chen J., Tsai M., Martin J.C., Smith C.D., Beckman J.S. (1992). Peroxynitrite-mediated tyrosine nitration catalyzed by superoxide dismutase. Arch. Biochem. Biophys..

[3] Frein D., Schildknecht S., Bachschmid M., Ullrich V. (2005). Redox regulation: a new challenge for pharmacology. Biochem. Pharmacol..

[4] Schildknecht S., Bachschmid M., Ullrich V. (2005). Peroxynitrite provides the peroxide tone for PGHS-2-dependent prostacyclin synthesis in vascular smooth muscle cells. FASEB J..

[5] Good P.F., Hsu A., Werner P., Perl D.P., Olanow C.W. (1998). Protein nitration in Parkinson's disease. J. Neuropathol. Exp. Neurol..

[6] Beckman J.S., Koppenol W.H. (1996). Nitric oxide, superoxide, and peroxynitrite: the good, the bad, and ugly. Am. J. Physiol..

[7] Smith M.A., Richey Harris P.L., Sayre L.M., Beckman J.S., Perry G. (1997). Widespread peroxynitrite-mediated damage in Alzheimer's disease. J. Neurosci..

[8] Reynolds M.R., Berry R.W., Binder L. (2007). Nitration in neurodegeneration: deciphering the "Hows" "nYs". Biochemistry.

[9] Schildknecht S., Karreman C., Daiber A., Zhao C., Hamacher J., Perlman D., Jung B., van der Loo B., O'Connor P., Leist M., Ullrich V., Bachschmid M.M. (2012). Autocatalytic nitration of prostaglandin endoperoxide synthase-2 by nitrite inhibits prostanoid formation in rat alveolar macrophages. Antioxidants Redox Signal..

[10] Zou M., Martin C., Ullrich V. (1997). Tyrosine nitration as a mechanism of selective inactivation of prostacyclin synthase by peroxynitrite. Biol. Chem..

[11] Yamakura F., Taka H., Fujimura T., Murayama K. (1998). Inactivation of human manganese-superoxide dismutase by peroxynitrite is caused by exclusive nitration of tyrosine 34 to 3-nitrotyrosine. J. Biol. Chem..

[12] Souza J.M., Giasson B., Chen Q., Lee V.M., Ischiropoulos H. (2000). Dityrosine cross-linking promotes formation of stable alpha-synuclein polymers. Implication of nitrative and oxidative stress in the pathogenesis of neurodegenerative synucleinopathies. J. Biol. Chem..

[13] Metanis N., Keinan E., Dawson P.E. (2010). Traceless ligation of cysteine peptides using selective deselenization. Angew Chem. Int. Ed. Engl..

[14] Muir T.W., Sondhi D., Cole P.A. (1998). Expressed protein ligation: a general method for protein engineering. Proc. Natl. Acad. Sci. U. S. A..

[15] Hejjaoui M., Butterfield S., Fauvet B., Vercruysse F., Cui J., Dikiy I., Prudent M., Olschewski D., Zhang Y., Eliezer D., Lashuel H.A. (2012). Elucidating the role of C-terminal post-translational modifications using protein semisynthesis strategies: α-synuclein phosphorylation at tyrosine 125. J. Am. Chem. Soc..

[16] Fauvet B., Butterfield S.M., Fuks J., Brik A., Lashuel H.A. (2013). One-pot total chemical synthesis of human α-synuclein. Chem. Commun..

[17] Burai R., Ait-Bouziad N., Chiki A., Lashuel H.A. (2015). Elucidating the role of site-specific nitration of α-synuclein in the pathogenesis of Parkinson's disease via protein semisynthesis and mutagenesis. J. Am. Chem. Soc..

[18] Xie J., Schultz P.G. (2005). An expanding genetic code. Methods.

[19] Wang L., Brock A., Herberich B., Schultz P.G. (2001). Expanding the genetic code of Escherichia coli. Science.

[20] Wang L., Schultz P.G. (2001). A general approach for the generation of orthogonal tRNAs. Chem. Biol. (Lond.).

[21] Yokoyama K., Uhlin U., Stubbe J. (2010). Site-specific incorporation of 3-nitrotyrosine as a probe of pKa perturbation of redox-active tyrosines in ribonucleotide reductase. J. Am. Chem. Soc..

[22] Normanly J., Kleina L.G., Masson J.M., Abelson J., Miller J.H. (1990). Construction of Escherichia coli amber suppressor tRNA genes. III. Determination of tRNA specificity. J. Mol. Biol..

[23] Wang L., Magliery T., Liu D., Schultz P. (2000). A new functional suppressor tRNA/Aminoacyl−tRNA synthetase pair for the in vivo in-corporation of unnatural amino acids into proteins. J. Am. Chem. Soc..

[24] Schildknecht S., Gerding H.R., Karreman C., Drescher M., Lashuel H.A., Outeiro T.F., Di Monte D.A., Leist M. (2013). Oxidative and nitrative alpha-synuclein modifications and proteostatic stress: implications for disease mechanisms and interventions in synucleinopathies. J. Neurochem..

[25] Uversky V.N., Yamin G., Munishkina L.A., Karymov M.A., Millett I.S., Doniach S., Lyubchenko Y.L., Fink A.L. (2005). Effects of nitration on the structure and aggregation of alpha-synuclein. Brain Res Mol Brain Res.

[26] Giasson B.I., Duda J.E., Murray I.V., Chen Q., Souza J.M., Hurtig H.I., Ischiropoulos H., Trojanowski J.Q., Lee V.M. (2000). Oxidative damage linked to neurodegeneration by selective alpha-synuclein nitration in synucleinopathy lesions. Science.

[27] Karreman C. (2002). AiO, combining DNA/protein programs and oligo-management. Bioinformatics.

[28] Young T.S., Ahmad I., Yin J.A., Schultz P.G. (2010). An enhanced system for unnatural amino acid mutagenesis in E. coli. J. Mol. Biol..

[29] Cooley R.B., Feldman J.L., Driggers C.M., Bundy T.A., Stokes A.L., Karplus P.A., Mehl R.A. (2014). Structural basis of improved second-generation 3-nitro-tyrosine tRNA synthetases. Biochemistry.

[30] Schildknecht S., Pape R., Müller N., Robotta M., Marquardt A., Bürkle A., Drescher M., Leist M. (2011). Neuroprotection by minocycline caused by direct and specific scavenging of peroxynitrite. J. Biol. Chem..

[31] Larsen R.A., Wilson M.M., Guss A.M., Metcalf W.W. (2002). Genetic analysis of pigment biosynthesis in Xanthobacter autotrophicus Py2 using a new, highly efficient transposon mutagenesis system that is functional in a wide varie-ty of bacteria. Arch. Microbiol..

[32] Wisastra R., Poelstra K., Bischoff R., Maarsingh H., Haisma H.J., Dekker F.J. (2011). Antibody-free detection of protein tyrosine nitration in tissue sections. Chembiochem.

[33] Lobstein J., Emrich C.A., Jeans C., Faulkner M., Riggs P., Berkmen M. (2012). SHuffle, a novel Escherichia coli protein expression strain capable of correctly folding disulfide bonded proteins in its cytoplasm. Microb. Cell Factories.

[34] Sokolovsky M., Riordan J.F., Vallee B.L. (1967). Conversion of 3-nitrotyrosine to 3-aminotyrosine in peptides and proteins. Biochem. Biophys. Res. Commun..

[35] Balabanli B., Kamisaki Y., Martin E., Murad F. (1999). Requirements for heme and thiols for the nonenzymatic modification of nitrotyrosine. Proc. Natl. Acad. Sci. U. S. A..

[36] Datsenko K.A., Wanner B.L. (2000). One-step inactivation of chromosomal genes in Escherichia coli K-12 using PCR products. Proc. Natl. Acad. Sci. U. S. A..

[37] Espey M.G., Xavier S., Thomas D.D., Miranda K.M., Wink D.A. (2002). Direct real-time evaluation of nitration with green fluorescent protein in solution and within human cells reveals the impact of nitrogen dioxide vs. peroxynitrite mechanisms. Proc. Natl. Acad. Sci. U. S. A..

[38] Neumann H., Hazen J.L., Weinstein J., Mehl R.A., Chin J.W. (2008). Genetically encoding protein oxidative damage. J. Am. Chem. Soc..

[39] Ferrer-Sueta G., Campolo N., Trujillo M., Bartesaghi S., Carballal S., Romero N., Alvarez B., Radi R. (2018). Biochemistry of peroxynitrite and protein tyrosine nitration. Chem. Rev..

[40] Bartesaghi S., Radi R. (2018). Fundamentals on the biochemistry of peroxynitrite and protein tyrosine nitration. Redox Biol.

[41] Radi R. (2018). Oxygen radicals, nitric oxide, and peroxynitrite: redox pathways in molecular medicine. Proc. Natl. Acad. Sci. U. S. A..

[42] Deeb R.S., Nuriel T., Cheung C., Summers B., Lamon B.D., Gross S.S., Hajjar D.P. (2013). Characterization of a cellular denitrase activity that reverses nitration of cyclooxygenase. Am. J. Physiol. Heart Circ. Physiol..

[43] Osoata G.O., Ito M., Elliot M., Hogg J., Barnes P.J., Ito K. (2012). Reduced denitration activity in peripheral lung of chronic obstructive pulmonary disease. Tanaffos.

[44] Léger C.L., Torres-Rasgado E., Fouret G., Carbonneau M.A. (2008). First evidence for an LDL- and HDL-associated nitratase activity that denitrates albumin-bound nitrotyrosine--physiological consequences. IUBMB Life.

[45] Kuo W.N., Kanadia R.N., Shanbhag V.P., Toro R. (1999). Denitration of peroxynitrite-treated proteins by 'protein nitratases' from rat brain and heart. Mol. Cell. Biochem..

[46] Bertoncini C.W., Jung Y.S., Fernandez C.O., Hoyer W., Griesinger C., Jovin T.M., Zweckstetter M. (2005). Release of long-range tertiary interactions potentiates aggregation of natively unstructured alpha-synuclein. Proc. Natl. Acad. Sci. U. S. A..

[47] Fallah M.A., Gerding H.R., Scheibe C., Drescher M., Karreman C., Schildknecht S., Leist M., Hauser K. (2017). Simultaneous IR-spectroscopic observation of α-synuclein, lipids, and solvent reveals an alternative membrane-induced oligomerization pathway. Chembiochem.

[48] Mirzaei H., Schieler J.L., Rochet J.C., Regnier F. (2006). Identification of rotenone-induced modifications in alpha-synuclein using affinity pull-down and tandem mass spectrome-try. Anal. Chem..

